# Selective Similarity Preference: People with a “Darker Core” Desire More Partner Similarity in Dark Traits but Not in Big Five

**DOI:** 10.3390/ejihpe16090128

**Published:** 2026-08-27

**Authors:** Hao Wu, Shanhong Luo, Whitney Wall, Karryll Winborne-Phillips

**Affiliations:** 1 School of Health Management, Guangzhou Medical University, Guangzhou 510180, China; 2016990010@gzhmu.edu.cn; 2 Department of Psychology, Fayetteville State University, 1200 Murchison Rd, Fayetteville, NC 28301, USA; wwall1@uncfsu.edu (W.W.); kwinborn@uncfsu.edu (K.W.-P.)

**Keywords:** dark traits, dark core, Big Five, assortative mating, similarity

## Abstract

Research on assortative mating has shown that couples share strong similarities in demographic and attitudinal variables, while the similarity in personality is generally weak. However, a recent meta-analysis indicated that couple similarity in dark traits is considerably greater than that in the Big Five. If these differential similarities are primarily due to active preference, we expect that (1) the desire for similarity in dark traits should be higher than that for the Big Five and (2) the strongest desire for partner similarity in dark traits should come from the “darkest” individuals (i.e., with the darkest core). Participants (*n* = 2465) rated themselves and their ideal partner in Dark Triad and Big Five traits. They also rated their own dark core. The results showed consistent evidence for desired similarity in dark traits; however, this similarity was no greater than that for the Big Five. Further, dark core generally moderated desired similarity on dark traits such that people with a darker core preferred stronger partner similarity in dark traits. This pattern was only observed for Agreeableness on the Big Five. A new explanation was proposed to reconcile these paradoxical findings concerning desired and actual similarity in dark traits in comparison to the Big Five.

## 1. Introduction

*Assortative mating* refers to the phenomenon that two partners in a romantic relationship tend to be systematically matched in their characteristics (e.g., Harper & Zietsch, 2025; Luo, 2017). Matching in similar characteristics is termed *positive assortment*, whereas matching in complementary or opposite characteristics is *negative assortment*. The large body of assortative mating literature has produced two general conclusions: (1) similarity is the rule while complementarity is an exception; and (2) the degree of similarity displays a hierarchy by the domain, with the strongest similarities occurring in demographic variables (e.g., age, SES, race), followed by attitudes and values, and finally personality characteristics displaying the weakest similarity (e.g., see Epstein & Guttman, 1984; Horwitz et al., 2023; Luo, 2017 for reviews).

The assortative mating research has recently been extended to the *dark traits* (e.g., Hudek-Knezevic et al., 2023; Kardum et al., 2022, 2024; Yu et al., 2020). The term “dark traits” represents a number of aversive traits that are linked to various maladaptive behaviors and negative outcomes (see Muris et al., 2017). A recent meta-analysis by Richards et al. (2024) on assortative mating in dark traits indicated that couples not only are substantially similar in dark traits, but that their similarity in dark traits (ranging from 0.22 to 0.35 with Mean = 0.27) is considerably greater than the similarity in the Big Five (ranging from 0.08 to 0.21 with Mean = 0.13). Why would couples be more similar in dark traits compared to general personality traits? Luo (2017) discussed four different mechanisms that may result in similarity in dark traits among couples. The most intuitive and sensible mechanism is people’s active preference/choice of a partner with similar “darkness” over a dissimilar one, known as the *active choice hypothesis*. The other three mechanisms are characterized more as artifacts and not due to individuals’ preferences. For example, *market operation* may lead to couple similarity in dark traits. Specifically, because dark traits are not socially desirable traits, people with dark traits may have to settle with a similarly “dark” partner even if they would have preferred “lighter” traits, due to the high competition for the more attractive “lighter” partners and their own lack of appeal because of their dark traits. *Social homogamy* could contribute to similarity in dark traits if people with similar dark characteristics frequent the same social groups and occasions (e.g., a gang) where they might meet their future partner. Finally, similarity may be due to *convergence*—partners simply grow to be similar in their “dark” personality over time. If observed similarity in dark traits is primarily due to active choice rather than an artifact from market operation, social homogamy, convergence, or other processes, then we would expect individuals to show a clear differential preference for higher partner similarity in dark traits than general personality traits such as the Big Five.

Furthermore, we argue that merely seeing evidence for desired similarity in dark traits would not be sufficient to validate the active choice hypothesis. That is because, for individuals who are “light” (i.e., have low scores on dark traits), any evidence for their similarity preference would be confounded with the evidence for positivity preference (i.e., a preference for generally positive characteristics which would include low scores on dark traits). In other words, “light” individuals’ preference for a similar, “light” partner could be due to positivity preference instead of a genuine desire for similarity. We reason if similarity is truly due to the active choice rather than a confound, the strongest support for desired similarity should come from those who are the “darkest” in personality; in other words, we expect dark core—the common core of dark traits (Moshagen et al., 2018) to moderate this “dark” similarity preference such that people with a “darker” core, compared to those less dark, would desire a partner with stronger similarity in dark traits.

### 1.1. Preference for Romantic Partner Similarity in Personality

Compared to the solid research on assortment patterns in actual couples, there has been much less direct examination on aspirational assortment patterns—whether there is similarity between self-ratings and ideal partner preferences on personality traits. Among the few studies that did examine this issue, some investigated attachment (e.g., Klohnen & Luo, 2003) and other personality characteristics (Watson et al., 2014), while most of this limited work focused on the Big Five (Botwin et al., 1997; Figueredo et al., 2006; Kleppestø et al., 2026; Watson et al., 2014). Their findings provide clear and consistent evidence for substantial preferred similarity in all Big Five traits, with the strongest similarity preference on Openness and Agreeableness, and lowest on Neuroticism (Botwin et al., 1997; Figueredo et al., 2006; Kleppestø et al., 2026; Watson et al., 2014). Watson et al. (2014) was the only study that explicitly compared desired and actual similarity in personality including the Big Five and a number of other personality characteristics relevant to mate preference. In two separate samples, they found that desired similarity was positively associated with actual similarity; however, desired similarity was noticeably stronger than actual similarity.

### 1.2. Romantic Partner Preferences in Terms of Dark Traits

Although “dark traits” is commonly used to describe a range of maladaptive traits, *Dark Triad*, first introduced by Paulhus and Williams (2002), specifically refers to three of the most prominent dark traits: *Narcissism* is characterized by qualities such as grandiosity, egotism, entitlement, and dominance. Individuals high on *Machiavellianism* are found to be highly manipulative, characterized by selfishness and a lack of a sense of morality. Individuals high on *psychopathy* tend to be highly impulsive and thrill-seeking while also demonstrating callousness, anxiety, and a lack of empathy. A meta-analysis on the research of dark traits by Muris et al. (2017) suggests that each of the dark traits is unique in their own right; on the other hand, there are high intercorrelations among the three Dark Triad traits, indicating that there is a considerable amount of commonality. Moshagen et al. (2018) proposed to conceptualize dark personality as a general, fluid dispositional Dark Factor which they named “dark core” with various dark traits as specific manifestations of this core. Dark core is “the tendency to maximize one’s individual utility—disregarding, accepting, or malevolently provoking disutility for others—accompanied by beliefs that serve as justifications” (Moshagen et al., 2018, p. 656).

A few studies have dived into dark traits in relation to mating preference or attraction. These studies showed that although dark traits are not highly desirable in long-term relationships, they could be attractive in instant attraction and short-term mating settings (e.g., Kesenheimer et al., 2026), especially narcissistic partners and to female attraction (e.g., Carter et al., 2014; Jauk et al., 2016; Jonason et al., 2011). Sexual evolution has been suggested as a potential explanation for this pattern: individuals with dark traits, particularly narcissistic males, may present themselves with some charming features such as confidence and boldness that are welcomed by women.

Most relevant to the current study is evidence for a preference for “dark” similarity in a romantic partner. Overall, the findings have been relatively consistent with a positive assortment preference. For example, Jonason et al. (2011) found that people high in Dark Triad tended to prefer partners with low levels of kindness, especially those high in psychopathy. Using experimental manipulations of partner’s dark traits, Jonason et al. (2015) found a strong indication for desired similarity in psychopathy, followed by Machiavellianism, but the support for desired similarity in narcissism was weak and inconsistent. The most direct evidence came from Kay’s (2021) study where two separate samples showed replicable evidence that people high on narcissism, Machiavellianism, and psychopathy were more willing to engage in a variety of relationships with a partner who also scored highly on narcissism, Machiavellianism, and psychopathy, respectively.

### 1.3. The Current Study

While previous research has shown evidence for desired similarity or aspirational positive assortment in dark traits, it is not sufficient to explain the difference in actual similarity observed in dark traits and the Big Five for two reasons. First, no study has directly compared the desired partner similarity in dark traits vs. Big Five. Thus it is unclear whether the apparent differential level of actual similarity in dark traits vs. Big Five is driven by a differential preference for similarity in these two domains. If the active choice hypothesis is true, we expect that people will desire stronger similarity in dark traits than in the Big Five; that is, there will be positive correlations between self and ideal partner’s dark traits (H1a); additionally, these correlations should be stronger than those for the Big Five (H1b). Second, none of the previous studies has explored individual differences in desired similarity. As the correlations between self and ideal partner ratings in personality are hardly perfect, clearly some people desire more similarity than others. If the active choice hypothesis was true, we expect that the strongest desire for similarity in dark traits will come from individuals with the “darkest” core; that is, dark core will moderate the association between self and ideal partner dark traits (H2). We explored these hypotheses in a large sample of young adults.

## 2. Method

### 2.1. Participant and Procedure

Participants for this study were 2465 college students from a relatively large, public university in southeastern U.S. The sample included 1859 women (75.4%), 546 men (22.2%), and 60 individuals (2.4%) who did not report their gender information. The average age was 19.30 years old (*SD* = 2.50 years). Participants were mostly Caucasian (82.6%), and Christian (61.7%). The vast majority (88.3%) of the sample had completed high school and some college education. In terms of sexual orientation, 64.1% of the sample indicated “only heterosexual”, 12.5 “mostly heterosexual”, 10.8% “bisexual”, 2.6% “mostly homosexual”, 8.2% “only homosexual”, and 1.8% did not respond to this question. About half (49.5%) of participants reported that they were currently single, while the other half were either in a committed relationship (34.8%) or casually dating someone (13.1%).

Participants were recruited from the subject pool at a large public university in the southeastern United States from 2020 to 2022. Students who consented to participate in the study were provided a web link to the online study. They responded to a series of measures concerning themselves and their preferred ideal partner. Upon completion, participants received one research credit for their voluntary participation in the study. The study received IRB approval at a local institution. While this study was not preregistered, we adopted an open science approach and all study materials and data are publicly available at https://osf.io/fjukq/files/osfstorage (accessed on 13 June 2026).

As the main objective of the current study was to test novel hypotheses regarding the moderating role of dark core on similarity preference for dark traits, we conducted a sensitivity analysis. Given an alpha level of 0.05, our sample size of 2465 was sufficient to detect an *R*^2^ increase as small as 0.003 with 80% power.

### 2.2. Measures

Only measures relevant to the purpose of the current study are described in detail below (see Table 1 for the alpha reliability of the measures reported in the study).

Demographic Questionnaire. The demographic questionnaire included information regarding participant gender, age, ethnicity, religion, relationship status, relationship length, education, income, etc.

Dark traits—self and ideal partner. To measure self dark traits, we used the 27-item Short Dark Triad (Jones & Paulhus, 2014) which includes three subscales: narcissism, Machiavellianism, and psychopathy, each containing nine items. Sample items are: “I know that I am special because everyone keeps telling me so” (narcissism); “Whatever it takes, you must get the important people on your side” (Machiavellianism); “I like to get revenge on authorities” (psychopathy). The items were rated on a 5-point Likert scale ranging from “Strongly Disagree” to “Strongly Agree.”

To measure ideal partner’s dark traits, we used the 12-item Dirty Dozen measure (Jonason & Webster, 2010) also with three subscales—narcissism, Machiavellianism, and psychopathy, each containing four items. Participants were asked to rate to what extent they prefer their ideal partner to have each of the characteristics. Sample items include: “wants others to admire him/herself” (narcissism); “manipulates others to get his/her way” (Machiavellianism); “is unconcerned with the morality of his/her actions” (psychopathy). Participants used a 5-point Likert scale which ranged from “Not at all” to “Very Much” to rate each item.

Dark core—self. The 16-item Dark Core measure (Moshagen et al., 2019) was used to assess participants’ identification with dark core. Sample items are “It is hard for me to see someone suffering” (reverse-scored), “I make a point of trying not to hurt others in pursuit of my goals”. A 5-point scale (ranging from strongly disagree to strongly agree) was used to indicate how each item was descriptive of participants themselves.

Big Five personality—self and ideal partner. We used the 30-item short-form Big Five Inventory-2-S (Soto & John, 2017) with five subscales: Extraversion, Agreeableness, Conscientiousness, Negative Emotionality, and Open-Mindedness. Each subscale contains six items. Sample items are: “acts as a leader” (Extraversion); “has a soft heart” (Agreeableness); “Is reliable” (Conscientiousness); “Tends to feel depressed, blue” (Negative Emotionality); “Is fascinated by art, music, or literature” (Openness). Participants used a 5-point Likert scale ranging from “Strongly Disagree” to “Strongly Agree” to indicate how well the statements represented the participant.

To measure the ideal partner’s Big Five, we used the 15-item extra short Big Five Inventory-2-XS (Soto & John, 2017) which also contains the same five subscales with three items for each subscale. The items were modified to target the participant’s ideal partner. Participants used a 5-point Likert scale which ranged from “Not at all” to “Very Much” to indicate how well each of the statements aligned with their ideal partner. 

## 3. Results

### 3.1. Preliminary Analyses

Table 1 presents the descriptive statistics and intercorrelations (Pearson correlations) for gender, age, and dark traits, and Big Five traits. All but three variables (age, Extraversion, Openness) showed significant gender differences, indicating that compared to women, men rated themselves higher on all dark traits and lower on Agreeableness, Conscientiousness, and Negative Emotionality. These gender differences ranged from small to medium in size. The dark traits had strong positive correlations with each other. This is especially true for Machiavellianism, psychopathy, and dark core as their intercorrelations were large in size, whereas correlations involving narcissism were medium. Dark traits had the strongest and most consistent correlations with Agreeableness, followed by Conscientiousness. It is clear that narcissism is the most desirable dark trait as it was positively correlated with socially desirable traits such Extraversion, Conscientiousness, and Openness and negatively correlated with the aversive trait Negative Emotionality. These gender difference and correlation findings are highly consistent with the previous literature on dark traits (e.g., Moshagen et al., 2018; Muris et al., 2017).

### 3.2. Primary Analyses

To test H1a—participants who score high on dark traits will also want their partner to be high on dark traits—we calculated the Pearson correlation between participants’ own scores of dark traits and their preferred ideal partner’s scores of dark traits (see Table 2). These correlational results showed that (1) there are significant, positive, moderately strong correlations between self and ideal partner on narcissism and Machiavellianism, as well as psychopathy. (2) Dark core had strong positive correlations with ideal partner’s narcissism, Machiavellianism, and psychopathy. (3) The correlations between self dark traits and ideal partner dark traits were all significantly positive and moderate in size. Overall, these correlations provided good and consistent support for H1a; that is, people did wish their partners to share a similarity in dark traits to a certain degree.

To test H1b—self-ideal partner similarity correlations for dark traits will be stronger than those for the Big Five—we first computed the self–ideal partner similarity correlations (Pearson correlation) on Big Five traits (see Table 3). All five correlations on the diagonal were statistically significant (*p* < 0.001) and ranged from medium to large in size. When we compared the Big Five self–ideal partner similarity correlations to the dark trait similarity correlations individually, two correlations from the Big Five traits (Agreeableness and Openness) were higher than the dark trait correlations and two were lower (Extraversion and Negative Emotionality), whereas the similarity correlation for Conscientiousness was similar in size to the dark trait correlations. Thus, the overall pattern was not consistent. To make this comparison clearer, we compared the average of these similarity correlations for dark traits and the Big Five. To do this, we Fisher-Z-transformed the Pearson correlations, computed the average of these transformed correlations for dark traits and the Big Five, and then completed the Fisher-Z-inverse transform for the averages. The desired similarity correlation average for dark traits was 0.33, whereas the correlation average for the Big Five was 0.35. Since the desired similarity in the Big Five was slightly higher than that in dark traits, which was in the opposite direction to the expectation in H1b, these results did not support H1b.

To test H2—participants with a “darker” core will desire stronger similarity in ideal partner dark traits—we conducted three sets of hierarchical regressions, each set containing three regressions. In the first set of regressions, ideal partner narcissism was the outcome, the moderator was self dark core, and the predictor was self narcissism, Machiavellianism, and psychopathy, respectively in each regression. Gender was the control variable in all regressions. The second set of regressions were the same as the first except the outcome was ideal partner Machiavellianism. In the last set, the outcome was ideal partner psychopathy. Prior to the regression analyses, all continuous predictors were standardized. We followed the same hierarchical procedure in all individual regressions: at Step 1 we entered participant gender as a control variable. At Step 2, we added the main predictor—the participants’ self dark trait (narcissism, Machiavellianism, or psychopathy) and the moderator—dark core. At Step 3, we added the interaction between the self dark trait (narcissism, Machiavellianism, or psychopathy) and dark core. This interaction term was created by multiplying the target self dark trait (narcissism, Machiavellianism, or psychopathy) and dark core. Standard regression coefficients for the predictors and control variables at Steps 2 and 3 as well as *R*^2^ are presented in Table 4. The multicollinearity diagnostics showed that tolerance scores in all regressions ranged from 0.86 to 1.00, suggesting minimal risks of collinearity. 

If H2 was true, we would expect the interactions between dark core and the individual self dark trait to be statistically significant in predicting each of the ideal partner dark traits. Out of the nine interaction effects, seven were statistically significant, all consistently sharing the same direction. The strongest interaction effects tended to involve the predictor of self psychopathy, while the only two interactions that failed to reach statistical significance both concerned predicting ideal partner narcissism.

**Figure 1:**
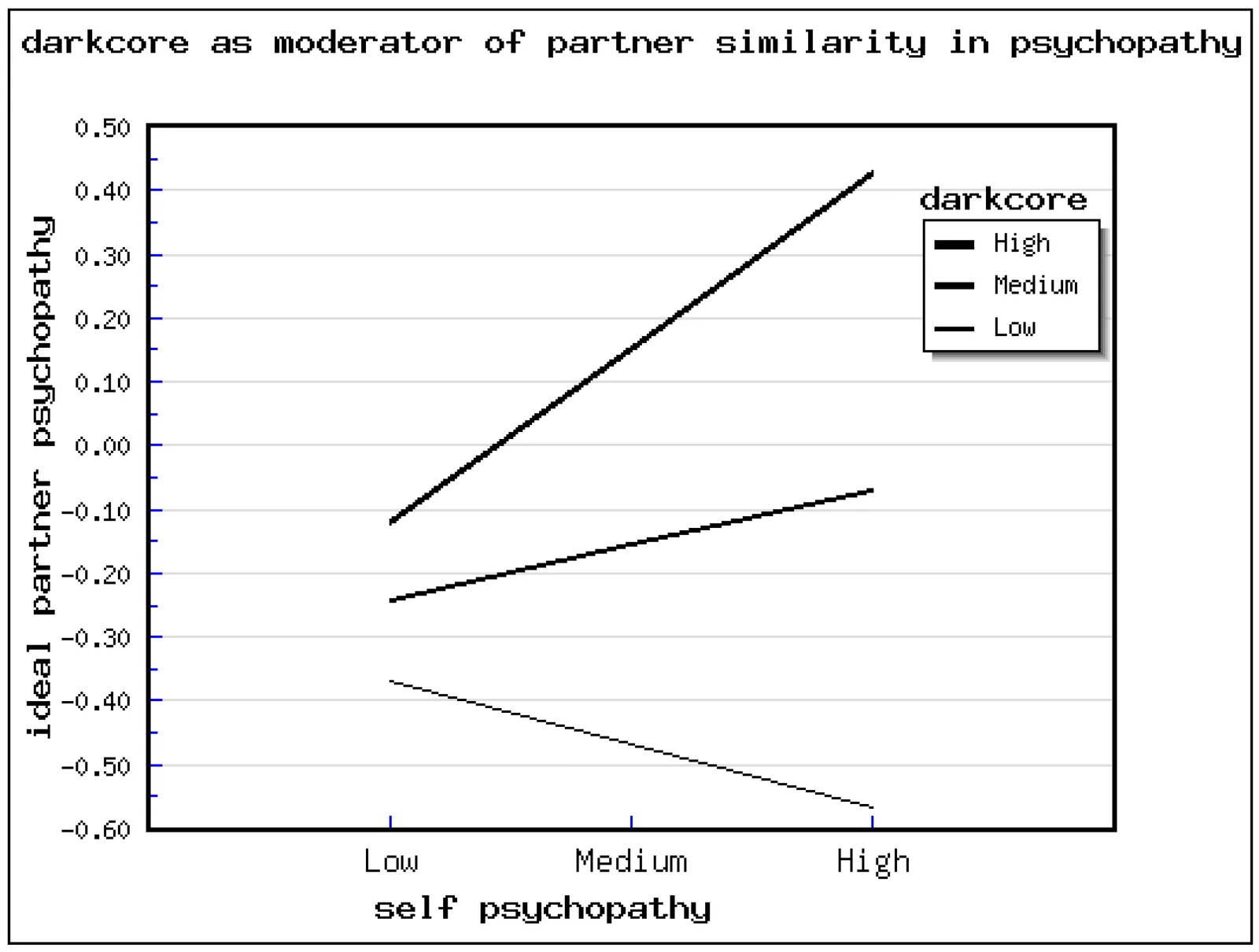
Dark core as a moderator of partner similarity in psychopathy.

To illustrate the nature of these similar interaction effects, we graphed the strongest interaction—dark core × psychopathy predicting ideal partner psychopathy (see Figure 1). Simple slope analyses indicated that for individuals with the highest dark core scores, self psychopathy was a strong, positive predictor of ideal partner psychopathy (B = 0.27, *p* < 0.001); for those with medium dark core scores, self psychopathy positively predicted ideal partner psychopathy with lesser strength (B = 0.07, *p* < 0.05); for those with the lowest dark core scores, self psychopathy negatively predicted ideal partner psychopathy (B = −0.10, *p* < 0.01). These slope analyses suggested that people with the darkest core desired the strongest similarity from their partner on dark traits and those with a medium dark core desire less of such similarity, while people with the “lightest” core desired the opposite or complementarity dark traits from their partner.

In terms of the effect size, the interaction term alone, when significant, was able to account for a range of 0.2% to 3.7% of the unique variance in ideal partner dark traits, which is considered small (Cohen et al., 2003). Taken together, these regression results provided good support for H2—dark core moderated the similarity preference effect on dark traits; specifically, for people with a “darker core”, they tend to show a stronger preference for partner similarity in dark traits. This trend was particularly strong in psychopathy and weakest in narcissism.

As a comparison, we then checked whether this moderation effect of dark core would generalize to non-dark traits. We conducted five similar hierarchical regressions for the Big Five. Specifically, we examined whether the interaction between dark core and each of the Big Five significantly predicted the corresponding ideal partner’s Big Five trait. The multicollinearity diagnostics showed that tolerance scores in all regressions ranged from 0.63 to 1.00, suggesting minimal risks of collinearity. As can be seen in Table 5, out of the five interaction effects, three did not reach statistical significance. One significant interaction (between dark core and Openness) was in the opposite direction to our expectation, suggesting the “darkest” individuals actually desired less dark similarity in Openness. The only significant interaction consistent with our expectation was between dark core and Agreeableness; however, the *R^2^* was lower than all of the *R*^2^s for dark traits. Moreover, as Agreeableness tends to correlate strongly with dark traits, this significant interaction basically aligned with H2. Overall, there was no strong evidence indicating that individuals with a darker core prefer more partner similarity in Big Five. Thus, our results suggest that people with a darker core do not just wish for more partner similarity in any personality traits but only in dark traits.

## 4. Discussion

Richards et al.’s (2024) meta-analysis indicated that the actual couple similarity correlations on dark traits are noticeably higher than those on the Big Five. The most plausible explanation for this difference in assortment strength is active preference and choice. Therefore, it is important to examine individuals’ partner similarity preferences in dark traits compared to the Big Five. If the active choice hypothesis can explain this result, we would expect two outcomes. First, people would desire stronger similarity in dark traits than in the Big Five. Findings from our large sample of young adults did show significant positive correlations between self-ratings and ideal partner ratings on dark traits, suggesting that people do desire partner similarity in dark traits. However, there was no indication that desired similarity in dark traits was higher than that in the Big Five, which was *not* consistent with the prediction based on the active choice hypothesis.

The second expectation based on the active choice hypothesis was that the strongest desire for similarity in dark traits would come from individuals with the “darkest” core; that is, dark core would moderate the association between self and ideal partner dark traits. Our moderator analyses showed that, with very few exceptions, dark core successfully moderated the desired similarity preference in dark traits, particularly for psychopathy and Machiavellianism. These moderation results suggested that for people who had the darkest core, they displayed a stronger preference for their partner to be similar to themselves on these dark traits (especially psychopathy and Machiavellianism), compared to those who scored in the middle range of dark core. Those with the lowest dark core actually showed evidence for “opposites attract” instead of similarity. Further, dark core generally failed to moderate the desired similarity in the Big Five in the same way it moderated the desired similarity in the dark traits (with the exception of Agreeableness which is strongly related to dark traits), suggesting that people with the darkest core do not just find any personality similarity appealing—they selectively desire similarity in dark traits. These moderation results were consistent with the active choice hypothesis.

Taking the correlation and the moderation results together, it appears that people do prefer partner similarity in dark traits; more importantly, this preferred similarity effect is primarily driven by people who are darker in core. Thus, the desire for similar dark traits seems genuine. Importantly, we also found that the desired similarity correlations obtained in the current study (ranging from 0.26 to 0.38) were comparable in size when compared to the actual similarity correlations in Richards et al.’s (2024) meta-analysis (ranging 0.22 to 0.37). This suggests that not only do people desire a partner similar to themselves in dark traits, they are also usually able to secure one.

The surprises came from the Big Five. Specifically, desired similarity correlations in the Big Five were not lower compared to those in dark traits as we had expected. This creates an apparent paradox—people desire the same level of similarity in the Big Five as in dark traits, but somehow through the matching process most people end up with a partner who is not particularly similar to themselves in these general personality traits—at least not to the degree they stated in their preferences. In contrast, evidently the desire for similarity in dark traits is maintained in the coupling process and realized in their actual partner.

Here we offer a post hoc explanation for this paradox. We suggest that the more rare or unique one’s characteristic or condition is, the more they would desire similarity in that characteristic or condition. This effect should be particularly strong for characteristics or conditions that are not socially desirable. For example, research on assortative mating in mental disorders and problem behaviors has shown that couples tend to have similar psychological problems; further, the similarity correlations in those domains are quite strong and substantially higher than the typical weak similarity correlations in personality (see Luo, 2017). We believe this is because sharing a unique feature with the most significant other in the world is likely to foster an especially strong sense of self-verification and affinity, more sustainable than sharing some common features that everybody else has as well (e.g., sharing the same nationality). This may explain why there is evidence for desired similarity but no substantive actual similarity in the Big Five. Because partner choice is a multi-faceted decision, it is likely that the desire for similarity in common traits like the Big Five gets trumped by other stronger desires, for example, the desire for a more stable income or a certain faith.

When individuals share some rare, unique features with their partner and these features happen to be undesirable and not easy to change (e.g., unappealing personality traits, psychological disorders, rare diseases, and certain physical defects), being able to share the same undesirable features with their closest partner may bring a good deal of comfort that could enable them to defy loneliness and low self-esteem that otherwise would occur. This may help to explain why the desired similarity correlation and the moderation effect were always stronger on Machiavellianism and psychopathy than narcissism in the current study—out of the three dark traits, narcissism is the most normal and desirable one (Muris et al., 2017). There may be other explanations for the weaker effect for narcissism. For example, narcissists may not strongly desire a partner who is also narcissistic because that might affect their “centeredness.” More generally, the “rareness” hypothesis may not be the only possible explanation for the differential findings between dark traits and the Big Five observed here as these two sets of traits differ in other aspects including the level of abstraction, social desirability, and measurement. Finally, it is important to note that the dark traits were conceptualized and measured at a subclinical level rather than as a pathological condition in the current study. It would be important to replicate these findings in clinical samples with individuals formally diagnosed with personality disorders such as narcissism or antisocial personality disorder. We hope future research will test this hypothesis about the moderating effects of the uniqueness and aversiveness of the features on desired as well as actual couple similarity.

This study was not without limitations. A major limitation is that while our sample was large in size, it was nevertheless a typical WEIRD sample (i.e., Western, educated, industrialized, rich, and democratic culture). Moreover, the majority of our participants were White, young, female college students who may hold differential ideal partner images compared to those who do not fall in these demographic categories. Another limitation is that while most measures in the study displayed acceptable to high reliability, the Big Five measure for ideal partners showed lower reliability due to its extreme short length. Given the exploratory nature of the current study, it would be useful for future studies to replicate the current findings in more diverse samples and measures with higher reliability for ideal partners.

Despite the limitations, the current study first proposed and provided support for “the darker want more similarity in darkness” hypothesis, which is in line with the active choice hypothesis of assortative mating. However, the current findings also indicated that desired similarities were about equal in dark traits and the Big Five, whereas the actual similarity in dark traits was considerably higher than that on the Big Five. This pattern was not consistent with the active choice hypothesis. As an attempt to solve this puzzle, we suggest that the preference for similarity in rare, unique features, particularly the undesirable ones, is likely to be strong, most genuine, and more likely to be actualized. We believe this “rareness” hypothesis would be useful to better understand the underlying mechanism of assortative mating and strongly encourage researchers to test it further in future research.

## Figures and Tables

**Table 1:** Descriptive statistics, alpha reliability, and intercorrelations of dark traits and Big Five traits.

					*r*									
		M	SD	Alpha	1	2	3	4	5	6	7	8	9	10
1.	Gender	-	-	-										
2.	Age	19.30	2.50	-	0.04									
3.	Dark core	1.94	0.55	0.82	0.12 ***	−0.03								
4.	Narcissism	2.82	0.61	0.67	0.06 ***	−0.04 *	0.27 ***							
5.	Machiavellianism	2.65	0.72	0.81	0.08 ***	−0.05 *	0.56 ***	0.32 ***						
6.	Psychopathy	2.05	0.65	0.75	0.11 ***	−0.01	0.69 ***	0.33 ***	0.54 ***					
7.	Extraversion	3.37	0.73	0.72	0.00	−0.01	0.05 *	0.51 ***	−0.01	0.11 ***				
8.	Agreeableness	3.89	0.66	0.73	−0.08 ***	0.01	−0.58 ***	−0.11 ***	−0.37 ***	−0.51 ***	0.06 **			
9.	Conscientiousness	3.53	0.71	0.73	−0.07 ***	0.04	−0.23 ***	0.07 **	−0.19 ***	−0.31 ***	0.23 ***	0.33 ***		
10.	Negative Emotionality	3.02	0.86	0.82	−0.11 ***	−0.05 *	−0.01	−0.25 ***	0.03	−0.01	−0.33 ***	−0.09 ***	−0.27 ***	
11.	Openness	3.73	0.71	0.74	0.02	0.04	−0.20 ***	0.10 ***	−0.07 ***	−0.06 **	0.09 ***	0.14 ***	−0.02	0.06 **

Note. *N* = 2275 to 2405. * *p* < 0.05 ** *p* < 0.01 *** *p* < 0.001.

**Table 2:** Correlations between self-ratings and ideal partner ratings on dark traits.

	Ideal Partner Rating		
Self-Rating	Narcissism	Machiavellianism	Psychopathy
Narcissism	0.26 ***	0.19 ***	0.14 ***
Machiavellianism	0.30 ***	0.34 ***	0.22 ***
Psychopathy	0.30 ***	0.45 ***	0.38 ***
Dark Core	0.30 ***	0.49 ***	0.44 ***

Note. *N* = 2283 to 2285. *** *p* < 0.001. Correlations between self and ideal partner ratings on the same dark trait are highlighted in bold and italic.

**Table 3:** Correlations between self-ratings and ideal partner ratings on Big Five traits.

	Ideal Partner Rating				
Self-Rating	Extraversion	Agreeableness	Conscientiousness	Negative Emotionality	Openness
Extraversion	0.22 ***	0.07 ***	0.08 ***	−0.10 ***	−0.00
Agreeableness	0.20 ***	0.49 ***	0.24 ***	−0.18 ***	0.07 ***
Conscientiousness	0.13 ***	0.21 ***	0.32 ***	−0.18 ***	−0.05 *
Negative emotionality	0.03	−0.01	−0.04	0.15 ***	0.06 **
Openness	0.07 ***	0.07 ***	0.06 **	−0.03	0.52 ***

Note. *N* = 2300. * *p* < 0.05 ** *p* < 0.01 *** *p* < 0.001. Correlations between self and ideal partner ratings on the same trait are highlighted in both and italic.

**Table 4:** Standard regression coefficients predicting ideal partner’s dark traits from gender, self dark traits, and dark core.

Predictor	Outcome					
	Partner Narcissism		Partner Mach		Partner Psychopathy	
	Step 2	Step 3	Step 2	Step 3	Step 2	Step 3
Gender	***0.02***	***0.02***	0.06 ***	0.06 ***	0.06 **	0.06 **
Dark core	0.26 ***	0.25 ***	0.47 ***	0.46 ***	0.43 ***	0.43 ***
Narcissism	0.19 ***	0.19 ***	0.05 **	0.06 ***	0.02	0.02
Dark core × Narcissism		***0.01***		0.06 **		0.04 *
*R*^2^ change	0.128 ***	***0.000***	0.249 ***	0.003 **	0.202 ***	0.002 *
Gender	0.03	0.03	0.06 ***	0.06 ***	0.06 **	0.06 **
Dark core	0.20 ***	0.20 ***	0.43 ***	0.42 ***	0.46 ***	0.45 ***
Machiavellianism	0.19 ***	0.19 ***	0.10 ***	0.10 ***	−0.04	−0.04
Dark core × Mach		−0.01		0.10 ***		0.06 ***
*R*^2^ change	0.121 ***	0.000	0.252 ***	0.009 ***	0.203 ***	0.004 ***
Gender	0.02	0.02	0.06 **	0.06 ***	0.06 **	0.06 **
Dark core	0.19 ***	0.18 ***	0.35 ***	0.31 ***	0.35 ***	0.31 ***
Psychopathy	0.17 ***	0.16 ***	0.20 ***	0.16 ***	0.13 ***	0.09 ***
Dark core × Psychopathy		0.04 *		0.18 ***		0.21 ***
*R*^2^ change	0.112 ***	0.002 *	0.267 ***	0.028 ***	0.211 ***	0.037 ***

Note. *N* = 2228 to 2230. * *p* < 0.05 ** *p* < 0.01 *** *p* < 0.001. Results for predicting ideal partner dark traits with the same self dark traits are highlighted in bold and italic.

**Table 5:** Standard regression coefficients predicting ideal partner’s Big Five traits from gender, self Big Five traits, and dark core.

Predictor	Outcome	
	Step 2	Step 3
	Ideal Partner Extraversion	
Gender	−0.09 ***	−0.09 ***
Dark core	−0.20 ***	−0.20 ***
Extraversion	0.23 ***	0.22 ***
Dark core × Extraversion		−0.02
*R*^2^ change	0.100 ***	0.000
	Ideal Partner Conscientiousness	
Gender	−0.02	−0.02
Dark core	−0.28 ***	−0.28 ***
Conscientiousness	0.25 ***	0.25 ***
Dark core × Conscientiousness		0.01
*R*^2^ change	0.178 ***	0.000
	Ideal Partner Agreeableness	
Gender	−0.04 *	−0.04 *
Dark core	−0.33 ***	−0.32 ***
Agreeableness	0.30 ***	0.30 ***
Dark core × Agreeableness		0.04 *
*R*^2^ change	0.317 ***	0.001 *
	Ideal Partner Openness	
Gender	−0.00	−0.00
Dark core	−0.04 *	−0.04 *
Openness	0.51 ***	0.51 ***
Dark core × Openness		−0.039 *
*R*^2^ change	0.271 ***	0.001 *
	Ideal Partner Negative Emotionality	
Gender	0.06 **	0.06 **
Dark core	0.27 ***	0.27 ***
Negative Emotionality	0.16 ***	0.16 ***
Dark core × Neg emotionality		−0.04
*R*^2^ change	0.103 ***	0.001

Note. *N* = 2241. * *p* < 0.05 ** *p* < 0.01 *** *p* < 0.001.

## Data Availability

Data associated with this paper can be found at https://osf.io/fjukq/files/osfstorage (accessed on 13 June 2026).
